# Factors associated with the work–self balance of nurses in an advanced medical center

**DOI:** 10.1002/pcn5.47

**Published:** 2022-09-18

**Authors:** Hiroaki Okayasu, Norio Sugawara, Yasushi Kawamata, Masataka Shinozaki, Keita Tokumitsu, Yoshiteru Sato, Aoi Sato, Yumiko Uchibori, Tomie Komatsu, Norio Yasui‐Furukori, Kazutaka Shimoda

**Affiliations:** ^1^ Department of Psychiatry Dokkyo Medical University School of Medicine Tochigi Japan; ^2^ Health Services Center for Students and Staff Dokkyo Medical University Tochigi Japan; ^3^ Department of Nursing Dokkyo Medical University Hospital Tochigi Japan

**Keywords:** cohabiting children, marriage, nurse, occupational stress, work, self balance

## Abstract

**Aim:**

Balancing between personal and working life of nurses is important to increase their job satisfaction and to continue their careers. Our purpose was to investigate the relationship between nurses and work–self balance (WSB) at different phases of life, such as age, marriage and raising children, and the occupational factors that influence WSB that can be used to improve the work environment for nurses.

**Methods:**

Using a self‐administered questionnaire, we asked about gender, age, marital status, presence of children, working hours, and night shift. Occupational stresses, including WSB, were assessed with the New Brief Job Stress Questionnaire (New BJSQ) and the Organizational Justice Questionnaire (OJQ). The total number of unmarried and married respondents was 819. We investigated whether marital status and cohabiting children make a difference in WSB in the three age groups (less than 30 years, 31–40 years, and more than 41 years) using a Mann–Whitney *U* test. In addition, we examined occupational factors affecting WSB using multiple regression analysis.

**Results:**

The value of WSB negative was significantly greater in the group of married persons than in the group of unmarried persons (*p* < 0.05) and was significantly greater in the group with cohabiting children than in the group without cohabiting children (*p* < 0.01) only in the group aged 31–40 years. Multiple regression analysis indicated that significant occupational factors affecting WSB differed by each age group.

**Conclusion:**

This survey showed that the factors and degree of WSB vary according to the generation and family environment of nurses.

## Highlights

- A survey of 819 nurses was conducted with a self‐administered questionnaire.
- Work–self balance (WSB) negative (WSB‐N) was larger for married nurses aged 31–40 years.
- WSB‐N was larger for nurses who were aged of 31–40 years and raising children.
- Occupational factors affecting WSB were different for each age group.

## INTRODUCTION

The balance between personal and professional life is a topical issue of increasing concern due to the intrusion of new technologies into personal life, overlapping work and family time, new organizational systems, and the changing nature of work.^1^ This problem is accentuated in professions with intense working hours and schedules.^2^ Especially, this is more pronounced in professions who require a strong workforce, such as nursing, because the demand for medical services is increasing and the roles required of nurses are also becoming more complex and diverse.^3^

Work–life balance (WLB) is a contemporary term for achieving balance between work and lifestyle ambitions. Although WLB is not well defined, the implication is that the two experiences (work and home life) should be in a state of equilibrium for nurses to be happy and healthy.^4^ WLB refers to an employee's satisfaction with their working life and the balance of time spent between work and one's private life.^4^, ^5^

Several studies have reported that nurses often lack balance between work and non‐work life and personal life.^4^, ^6^ It has been reported that most nurses have felt that their WLB has been out of balance with the proportion of working greater than the proportion of personal life.^5^

In addition, it has been reported that nurses who exhibited less striving for WLB behavior showed higher intentions to leave and that securing a comfortable WLB would reduce the nurses' desire to quit their work.^7^ The shortage of nurses is a serious problem potentially, as the turnover rate of nurses is high, and many nurses do not return to work in Japan.^8^ Therefore, it is important for active nurses to stay in their jobs and continue their careers. One of the ways to achieve this is to improve WLB.

In Japan, WLB often refers to workers balancing work and nonwork activities, such as family, in each stage of their professional life. However, we conducted a survey on work–self balance (WSB) that also considered single workers rather than one on WLB that focused on families because the number of unmarried workers is increasing across all age groups in Japan.^9^

There are few previous studies comparing WSB between single and married people and WSB with and without children living together.

Here, we investigated the relationship between nurses and WSB at different phases of life, such as age, marriage, and raising children, and the occupational factors that influence WSB that can be used to improve the work environment for nurses working in a university hospital, which is an organization that provides advanced medical technology.

## METHOD

### Participants

A cross‐sectional survey of 1063 nurses working at Dokkyo Medical University Hospital was conducted from the end of July to the beginning of August 2020 using a self‐administered questionnaire. The total number of responses was 844 (79.4%).

The total number of unmarried and married respondents (those who answered 0 or 1 for the marital status item) was 819 (459 unmarried and 360 married) of the 844 valid responses received (Table 1). All the nurses at this hospital were shift workers.

**Table 1:** Demographic data of married and unmarried participants

Age	Number of unmarried persons (number of men) mean age (years) ± SD	Number of married persons (number of men)		Total number (number of men) mean age (years) ± SD
		Without cohabiting children, mean age (years) ± SD	With cohabiting children, mean age (years) ± SD	
Less than 30 years old	305	35	36	376
	(28)	(10)	(12)	(50)
	24.7 ± 2.43	27.1 ± 2.22	28.2 ± 1.71	25.3 ± 2.63
From 31 to 40 years old	90	27	105	222
	(11)	(6)	(23)	(40)
	34.9 ± 3.17	33.4 ± 2.41	35.9 ± 2.83	35.2 ± 3.03
More than 41 years old	64	22	135	221
	(2)	(1)	(9)	(12)
	48.4 ± 5.40	51.5 ± 5.93	46.9 ± 4.44	47.8 ± 5.06
Total	459	84	276	819
	(41)	(17)	(44)	(102)
	30.0 ± 8.97	33.5 ± 10.5	40.3 ± 7.8	34.0 ± 9.97

### Variables

#### Sociodemographic and employment characteristics

As for sociodemographic and employment characteristics (items related to individual factors), we asked about gender, age, marital status (never married, married, or other), presence of children living together, working hours, and night shift. Gender, marital status, presence of children living together, and night shift were used as categorical variables.

#### Comprehensive psychological distress

The participants' comprehensive psychological distress was assessed with the Kessler 6 (K6).^10^, ^11^ The K6 comprises six questions pertaining to the assessment of psychological distress, and higher K6 values indicate higher levels of psychological distress. In line with the recommended K6 cutoff point, any participants with total scores greater than 13 were defined as having severe psychological distress.^12^

#### Assessment of WSB and other occupational stresses based on the New Brief Job Stress Questionnaire

In this study, occupational stresses of nurses were assessed with the New Brief Job Stress Questionnaire (New BJSQ), which has been widely used and is established as a method for assessing job stress in Japan.^13^

The occupational stresses include quantitative job overload, qualitative job overload, job control, interpersonal conflict, emotional demands, role conflict, role clarity, career opportunity, predictability, supervisor support, coworker support, support from family and friends, workplace harassment, workplace social capital scales and WSB taken from the New BJSQ.

WSB has two indicators: positive and negative. WSB positive (WSB‐P) is defined as being energized by work and using what you learn at work to enhance your life, while SB negative (WSB‐N) is defined as thinking only about work schedule and work, and not fulfilling one's life.

Responses were made on a four‐point Likert scale: 1 = *Very much so*, 2 = *Moderately so*, 3 = *Somewhat*, and 4 = *Not at all*. For the item about support from superiors, co‐workers, and family/friends, the scale was as follows: 1 = *Extremely*, 2 = *Very much*, 3 = *Somewhat*, and 4 = *Not at all*. For each scale, there were two or three sub‐questions, and the average value divided by the sum of the scores obtained from each question by the number of items was used as the number of scores for each scale. The scale scores for any scale were transformed to show that a high score indicates a desirable state.

#### Questionnaire for organizational justice based on the Organizational Justice Questionnaire

The participating employees' perceptions of fairness were measured using the Organizational Justice Questionnaire (OJQ).^14^ The Japanese version of the OJQ is based on the scale by Elovainio et al.,^15^ which is a modified version of the original scale developed by Moorman.^16^ The OJQ consists of a seven‐item Procedural Justice subscale and a six‐item Interactional Justice subscale. All items of both subscales were rated on a five‐point Likert scale ranging from 1 (*strongly disagree*) to 5 (*strongly agree*), and higher scores indicated higher levels of organizational justice.

### Statistical analysis

#### Comparison of WSB by marital status in each generation

To compare the WSB in each generation, the data were divided into three groups according to age (0: less than 30 years, 1: 31–40 years, 2: more than 41 years). In addition, the data of each group were divided into unmarried and married, and Mann–Whitney *U* test was conducted for comparison of the difference in WSB according to marital status.

#### Comparison of WSB by presence or absence of cohabiting children in each generation

After dividing the participants into three groups by age as above, we divided them into two groups by the presence or absence of cohabiting children. The Mann–Whitney *U* test was conducted for comparison of the difference in WSB according to the presence or absence of cohabiting children.

#### Occupational factors affecting WSB

To examine occupational factors affecting WSB in each age group, multiple regression analysis was conducted with the value of WSB as the dependent variable and basic items (sex, age, job description, working hours, availability of night shift) and values of K6 and occupational factors obtained from the BJSQ and OJQ as independent variables.

All the analyses were conducted using SPSS Statistics Version 27.0 statistical software, and the significance level was set to 0.05 (two‐tailed test).

## RESULTS

Comparing the WSB of unmarried and married persons in each age group, the value of WSB‐N was significantly larger in the group of married persons than in the group of unmarried persons only in the 31–40‐years age group (*p* < 0.05). In other age groups, there was no significant difference in WSB‐N. There was no significant difference in WSB‐P between married and unmarried persons in any of the age groups (Table 2).

**Table 2:** Comparison about work–self balance between unmarried persons and married persons by age

Age	Work–self balance	Unmarried, mean ± SD	Married, mean ± SD	*p* Value
All ages	Work–self balance positive	2.10 ± 0.69	2.09 ± 0.73	0.93
	Work–self balance negative	2.24 ± 0.80	2.32 ± 0.78	0.22
Less than 30 years	Work–self balance, positive	2.11 ± 0.67	2.17 ± 0.73	0.47
	Work–self balance, negative	2.29 ± 0.80	2.33 ± 0.74	0.61
From 31 to 40 years	Work–self balance, positive	2.03 ± 0.74	2.09 ± 0.76	0.16
	Work–self balance, negative	2.06 ± 0.76	2.36 ± 0.79	0.010*
More than 41 years	Work–self balance, positive	2.13 ± 0.66	2.06 ± 0.70	0.52
	Work–self balance, negative	2.26 ± 0.85	2.28 ± 0.81	0.86

* *p* < 0.05.

Comparing the WSB between the groups with and without cohabiting children in each age group, WSB‐N was significantly greater in the group with cohabiting children than in the group without cohabiting children only in the group aged 31–40 years. In other age groups (*p* < 0.01), there was no significant difference in WSB‐N. There was no significant difference in WSB‐P between those with and without cohabiting children in any of the age groups (Table 3).

**Table 3:** Comparison of work–self balance with and without cohabiting child

Age	Work–self balance	Without cohabiting child, mean ± SD	With cohabiting child, mean ± SD	*p* Value
All ages	Work–self balance, positive	2.12 ± 0.69	2.09 ± 0.73	0.54
	Work–self balance, negative	2.24 ± 0.80	2.33 ± 0.78	0.17
Less than 30 years	Work–self balance, positive	2.13 ± 0.69	2.14 ± 0.79	0.90
	Work–self balance, negative	2.29 ± 0.79	2.28 ± 0.74	0.85
From 31 to 40 years	Work–self balance, positive	2.05 ± 0.72	2.11 ± 0.77	0.56
	Work–self balance, negative	2.06 ± 0.75	2.41 ± 0.77	0.0016**
More than 41 years	Work–self balance, positive	2.15 ± 0.69	2.05 ± 0.67	0.28
	Work–self balance, negative	2.28 ± 0.88	2.28 ± 0.79	0.95

** *p* < 0.01.

The results of multiple regression analysis to determine the occupational factors that affect WSB in each age group were as follows.

In the group under 30 years old, age (*p* < 0.05), job control (*p* < 0.01), career opportunity (*p* < 0.01), and predictability (*p* < 0.001) were positively significant relations for WSB‐P. On the other hand, K6 score (*p* < 0.05) and role clarity (*p* < 0.001) were negatively significant relations for WSB‐P. In the group from 31 to 40 years old, emotional demands (*p* < 0.05), career opportunity (*p* < 0.01), predictability (*p* < 0.001), and procedural justice (*p* < 0.01) were positively significant relations for WSB‐P. On the other hand, K6 score (*p* < 0.01), role conflict (*p* < 0.05), and support from family and friends (*p* < 0.05) were negatively significant relations for WSB‐P. In the group more than 41 years old, career opportunity (*p* < 0.01) and predictability (*p* < 0.01) were positively significant relations for WSB‐P (Table 4).

**Table 4:** Occupational factors affecting work–self balance positive by age

	Work–self balance positive		
	Less than 30 years	From 31 to 40 years	More than 41 years
	Forced entry	Forced entry	Forced entry
	Partial regression coefficient (95% CI)	Partial regression coefficient (95% CI)	Partial regression coefficient (95% CI)
Sex	−0.026(−0.20 to 0.15)	0.049 (−0.18 to 0.28)	−0.087 (−0.50 to 0.33)
Age	0.027 (−0.00034 to 0.053)*	−0.019 (−0.048 to 0.010)	0.016 (−0.0027 to 0.034)
Working hours	−0.036 (−0.097 to 0.025)	−0.039 (‐0.13 to 0.052)	0.024 (−0.069 to 0.12)
Availability of night shift	−0.031 (−0.30 to 0.24)	0.21 (−0.024 to 0.45)	−0.23 (−0.46 to 0.0025)
K6 score	−0.013 (−0.025 to −0.00053)*	−0.029 (−0.048 to −0.010)**	−0.011 (−0.032 to 0.011)
Quantitative job overload	0.062 (−0.089 to 0.21)	−0.035 (−0.25 to 0.18)	−0.026 (−0.27 to 0.22)
Qualitative job overload	0.0036 (−0.15 to 0.16)	0.089 (−0.15 to 0.33)	0.16 (−0.10 to 0.41)
Job control	0.21 (0.095 to 0.33)***	0.047 (−0.13 to 0.22)	−0.0052 (−0.17 to 0.16)
Interpersonal conflict	0.077 (−0.085 to 0.24)	−0.15 (−0.36 to 0.058)	0.013 (−0.21 to 0.23)
Emotional demands	0.058 (−0.070 to 0.19)	0.23 (0.044 to 0.41)*	0.0074 (−0.18 to 0.20)
Role conflict	−0.048 (−0.19 to 0.091)	−0.28 (−0.49 to −0.075)**	0.024 (−0.18 to 0.22)
Role clarity	−0.29 (−0.45 to −0.13)***	−0.038 (−0.27 to 0.19)	−0.077 (−0.31 to 0.15)
Career opportunity	0.23 (0.095 −0.37)**	0.28 (0.081 to 0.48)**	0.32 (0.12 to 0.52)**
Predictability	0.40 (0.28 to 0.52)***	0.39 (0.24 to 0.54)***	0.28 (0.11 to 0.45)**
Supervisor support	0.038 (−0.099 to 0.18)	0.025 (−0.18 to 0.23)	0.16 (−0.048 to 0.36)
Coworker support	−0.0051 (−0.12 to 0.11)	0.061 (−0.12 to 0.24)	−0.041 (−0.22 to 0.13)
Support from family and friends	−0.086 (−0.19 to −0.021)	−0.14 (−0.27 to 0.0081)*	−0.022 (−0.16 to 0.12)
Workplace harassment	−0.096 (−0.20 to 0.0063)	−0.0082 (−0.14 to 0.12)	−0.034 (−0.17 to 0.10)
Workplace social capital	0.031 (−0.096 to 0.16)	−0.077 (−0.25 to 0.10)	0.14 (−0.066 to 0.34)
Procedural justice	0.077 (−0.027 to 0.18)	0.22 (0.071 to 0.38)**	0.56 (−0.11 to 0.22)
Interactional justice	0.031 (−0.070 to 0.13)	0.082 (−0.056 to 0.22)	−0.12 (−0.27 to 0.037)

* *p* < 0.05.

** *p* < 0.001;

*** *p* < 0.001.

In the group under 30 years old, emotional demands (*p* < 0.001) were positively significant relations for WSB‐N, while K6 score (*p* < 0.001) and predictability (*p* < 0.05) were negatively significant relations for WSB‐N. In the group from 31 to 40 years old, sex (*p* < 0.05), age (*p* < 0.05), quantitative job overload (*p* < 0.01), career opportunity (*p* < 0.05), supervisor support (*p* < 0.01), and workplace harassment (*p* < 0.05) were positively significant relations for WSB‐N, while K6 score (*p* < 0.001) was a negatively significant relation for WSB‐N. In the group over 41 years old, age (*p* < 0.001), job control (*p* < 0.05), and career opportunity (*p* < 0.05) were positively significant relations for WSB‐N, while K6 score (*p* < 0.01) and role clarity (*p* < 0.01) were negatively significant relations for WSB‐N (Table 5).

**Table 5:** Occupational factors affecting work–self balance negative by age

	Work–self balance negative		
	Less than 30 years old	From 31 to 40 years old	More than 41 years old
	Forced entry	Forced entry	Forced entry
	Partial regression coefficient (95% CI)	Partial regression coefficient (95% CI)	Partial regression coefficient (95% CI)
Sex	0.035 (−0.16 to 0.23)	0.26 (0.026 to 0.50)*	−0.27 (−0.68 to 0.14)
Age	0.0049 (−0.024 to 0.034)	0.031 (0.00068 to 0.062)*	0.027 (−0.0084 to 0.045)**
Working hours	−0.064 (−0.13 to 0.0038)	−0.033 (−0.13 to 0.062)	−0.012 (−0.10 to 0.080)
Availability of night shift	0.010 (−0.29 to 0.31)	−0.077 (−0.32 to 0.17)	−0.011 (−0.24 to 0.22)
K6 score	−0.026 (−0.040 to −0.013)***	−0.047 (−0.067 to −0.027)***	−0.029 (−0.051 to −0.0078)**
Quantitative job overload	0.13 (−0.037 to 0.30)	0.37 (0.15 to 0.60)**	0.51 (0.26 to 0.75)***
Qualitative job overload	0.16 (−0.017 to 0.33)	0.021 (−0.23 to 0.28)	−0.042 (−0.29 to 0.21)
Job control	0.12 (−0.016 to 0.25)	−0.061 (−0.24 to 0.12)	0.31 (0.15 to 0.48)***
Interpersonal conflict	0.084 (−0.096 to 0.27)	−0.018 (−0.24 to 0.20)	0.022 (−0.19 to 0.24)
Emotional demands	0.25 (0.11 to 0.39)***	0.041 (−0.15 to 0.23)	−0.0090 (−0.20 to 0.18)
Role conflict	0.22 (0.066 to 0.37)	0.18 (−0.038 to −0.39)	0.18 (−0.017 to 0.38)
Role clarity	0.027 (−0.15 to 0.20)	0.10 (−0.14 to 0.34)	−0.36 (−0.59 to −0.14)**
Career opportunity	0.064 (−0.092 to −0.22)	0.21 (0.0055 to 0.42)*	0.23 (0.026 to 0.43)*
Predictability	−0.15 (−0.29 to −0.012)*	−0.15 (−0.31 to 0.0046)	0.032 (−0.14 to 0.20)
Supervisor support	0.093 (−0.061 to 0.25)	0.24 (0.021 to 0.46)*	−0.075 (−0.28 to 0.13)
Coworker support	−0.094 (−0.22 to 0.030)	−0.093 (−0.28 to 0.095)	−0.079 (−0.25 to 0.095)
Support from family and friends	0.071 (−0.048 to −0.19)	0.062 (−0.075 to 0.20)	−0.0077 (−0.15 to 0.13)
Workplace harassment	−0.0077 (−0.12 to 0.11)	0.14 (0.0086 to 0.28) *	0.076 (−0.061 to 0.21)
Workplace social capital	−0.072 (−0.21 to 0.069)	−0.16 (−0.35 to 0.021)	−0.12(−0.32 to 0.077)
Procedural justice	−0.040 (−0.16 to 0.077)	0.065 (−0.096 to 0.23)	0.071 (−0.092 to 0.23)
Interactional justice	0.047 (−0.066 to 0.16)	−0.073 (−0.22 to 0.071)	−0.022 (−0.17 to 0.13)

* *p* < 0.05.

** *p* < 0.001;

*** *p* < 0.001.

## DISCUSSION

In this study, in the age group of 31–40 years, the value of WSB‐N in the married group was significantly higher than that in the unmarried group, and in the age group of 31–40 years, the value of WSB‐N in the group with children living together was significantly higher than that in the group without children living together.

Nursing is a service‐oriented occupation that requires emotional labor, observing and assisting patients and their families, and working in shifts, which can easily disrupt the rhythm of life and cause a lot of stress. We tend to focus on the negative aspects, such as the difficulty of balancing work and family, because there are reports that work–family conflict (WFC) affects job satisfaction and the formation of attachment to children.^17^, ^18^ However, Sullivan et al. reported that married surgery residents were most likely to look forward to work, and report happiness at work and a good program fit, and surgery residents with children more frequently looked forward to work, were happy at work, and reported a good program fit, but had strain on family life, and worried about future finances.^19^ Although it was not possible to make direct comparisons between surgical residents and nurses because of the differences in their duties and work patterns, we thought that being married and having children might make it possible to have a positive experience as a medical professional, such as a doctor or nurse.

Furthermore, it has been reported that “buffering factors,” such as social support from superiors, colleagues, and family members, can act as buffers to the occurrence of stress reactions.^20^ Okada et al. measured the mental health of nurses who were divided into two groups according to whether they had ever left their jobs and showed that the group without job separation tended to have higher mental health than the group with job separation; they also reported that those with spouses had higher mental health than those without spouses.^21^ It could be predicted that nurses who are able to continue working without leaving their jobs even after experiencing major life events, such as marriage, are better able to cope with various stressors. The start of a new life, such as marriage and child rearing, among nurses aged 31–40 years might rather maintain a sense of fulfillment in their private time, and the value of WSB‐N rises, that is, they do not only think about work.

Life events, such as marriage and child rearing, may be acting as buffering factors rather than negative stressors. Yaktin et al. investigated the effect of personal characteristics and job satisfaction among nurses and found that married nurses had systematically higher job satisfaction than unmarried nurses, although the difference was not significant.^22^

On the other hand, in the generation of more than 41 years old, less intervention in child rearing due to the growth of children living with them might result in less fulfillment in terms of private life, and conversely, the psychological burden of assuming positions of increasing responsibility, such as managerial positions, might eliminate the significant difference due to marriage and the presence of children living with them.

In this study, both WSB‐P and WSB‐N were shown to decrease with greater psychological distress in the under‐30s group and the 31–40‐year‐old group significantly. In the generation over 40 years old, WSB‐N was shown to decrease significantly with increasing psychological stress. On the other hand, there was no significant difference in WSB‐P, but there was a tendency for WSB‐P to decrease with increasing psychological stress. Bilodeau et al. reported that the presence of WFC is associated with psychological distress.^23^ The presence of psychological distress is a factor that interferes with WSB even among nurses working in university hospitals, so it was thought that consideration should be given to reducing psychological distress.

Regarding occupational factors affecting WSB, career opportunity and predictability were positively associated with WSB‐P in all age groups in this study. Malinauskiene et al. suggested that the key factors that cause occupational stress should be identified, and the problem solved in the workplace.^24^ It was thought that the organization could better improve the WSB of nurses of all ages by providing individual nurses with a certain amount of work, but also by providing them with guidance on the direction of their work and preparing them with opportunities for growth.

It is also important to note that there are different factors affecting WSB in different age groups.

Wepfer et al. reported that work demands increased among people with cohabiting young children due to the overlap of career development and child‐rearing years, that WLB is highest among people aged 55 or older when their children have grown up and left home, and that WLB is higher among families with cohabiting young children who work part‐time than among those who work full‐time. Therefore, they considered the demands of work and the experience of WLB to be influenced by different life stages.^25^

In our study, in the younger generation (under 30s), age, job control, and career opportunity were positively related to WSB‐P and emotional demands were positively related to WSB‐N. Predictability was positively related to WSB‐P, but negatively related to WSB‐N. The results were contradictory. It has been reported that increased work demands increased the level of work–life imbalance among nurse physicians, while job control had a positive effect on balancing work and personal life.^26^ The younger generation could feel that it is better to be able to work at their own pace and to have less of a mental burden than a physical workload. On the other hand, there could be an aspect of being dissatisfied with the clarity of one's work role, as they feel that they continue to do what their superiors tell them to do in a rambling manner. Being able to predict what needs to be done at work is good, but they could also feel concern about a gradual increase in their workload as they become accustomed to their work.

In our study, the sex difference was only significant in the 31–40‐year‐old generation, indicating that being male is associated with WSB‐N. This could suggest that even if being married and having children increases job satisfaction, as discussed above, women still bear a greater burden for childcare than men in Japan.

In terms of work control, the younger generation of less than 30 years old could have the desire to be able to work at their own pace, but when they become middle managers over the age of 31, the workload increases, and they would rather not be able to control their work. At the age of 41 and above, senior management positions make it easier for them to reflect their own opinions in the workplace, which in turn makes it easier for them to take control of their work again, which could have an impact on WSB.

In the generation over 31 years old, less quantitative workload was positively related to WSB‐N, while in the generation under 30 years old, less emotional demands were positively related to WSB‐N.

This could indicate that in the younger generation who have just started working, less mental strain is better for WSB than physical workload, while in nurses over 31 years old, they might simply consider less physical workload to be better for WSB.

In the middle age group (31–40 years old), less emotional demands and higher procedural justice were positively related to WSB‐P, and male sex, high age, less quantitative overload, less supervisor support, and less workplace harassment were positively associated with WSB‐N. On the other hand, less role clarity and more support from family and friends were negatively related to WSB‐P. This could mean that many nurses in middle management positions have a heavy workload, and although they have support from family and friends, they do not feel it is sufficient. Rather, they could feel more secure in performing their duties and spend less time worrying about their work outside of work hours because they have appropriate, noncoercive support from their supervisors and fairness in decision‐making in the workplace.

It has been reported that organizational justice climate weakened the adverse effect of WFC on depressive symptoms and the buffering effects of procedural and distributive justice climate in the association between WFC and depressive symptoms depended on family flexibility.^27^

In nurses aged more than 40 years old, high age, less workload, and more control over work were positively related to WSB‐N. On the other hand, role clarity was negatively related to WSB‐N. Many nurses over 41 years old are in managerial positions, and they might spend a lot of time thinking about their work as managers even outside of work because their roles as managers are clear and they know their work.

It has been reported that being able choose the order in which one will perform tasks, being able to work at one's own pace and using one's own methods, being consulted about objectives that affect one's work, being able to influence the decisions that are important, or having the opportunity to apply one's own ideas at work, among other discretionary acts, cushions the negative effect that the psychological pressure of nursing work exerts on WLB.^28^ Therefore, it was thought that hospital administrators need to take different actions for each age group to improve the WSB of nurses.

### Limitation

There are several limitations to this study. First, the study was conducted in a single facility, and therefore, there is a bias in terms of the differences between the type of employment and work environment provided by this facility and that of the general population. Second, because the research design was cross‐sectional, it was not possible to identify causal relationships. Longitudinal studies are needed in the future to confirm this. Third, this study lacks important data, such as income and presence of physical and mental illness. Third, we did not include divorced persons in our study. This is why we do not know whether all the respondents in the “other” category were divorced, since we used the questionnaire to identify marital status as “unmarried,” “married,” or “other.” Finally, we did not include job position in our analysis because we thought there was some collinearity between age and job position, given the tendency for nurses to be promoted to higher positions as they grow older in Japan.

## CONCLUSION

This survey showed that the factors and degree of WSB vary according to the generation and family environment of nurses in an advanced medical center. It was thought that hospital administrators need to understand the WSB of nurses and take steps to alleviate occupational stress and motivate each generation differently to increase it.

## AUTHOR CONTRIBUTIONS

All authors made a significant contribution to the work reported, whether that is in the conception, study design, execution, acquisition of data, analysis and interpretation, or all these areas; took part in drafting, revising, or critically reviewing the article; gave final approval of the version to be published; have agreed on the journal to which the article has been submitted; and agree to be accountable for all aspects of the work.

## CONFLICT OF INTEREST

The authors declare no conflict of interest.

## ETHICS APPROVAL STATEMENT

This protocol for this study was approved by the Bioethics Committee of Dokkyo Medical University (approval number: 29112) and it complies with the Declaration of Helsinki and the Japanese Ethical Guidelines for Medical and Health Research Involving Human Subjects.

## PATIENT CONSENT STATEMENT

N/A

## Data Availability

The datasets supporting the conclusions of the current study are available from the corresponding author on reasonable request and if approved by the Bioethics Committee of Dokkyo Medical University.
